# After 50 Years of Heart Transplants: What Does the Next 50 Years Hold for Cardiovascular Medicine? A Perspective From the International Society for Applied Cardiovascular Biology

**DOI:** 10.3389/fcvm.2019.00008

**Published:** 2019-02-14

**Authors:** Joshua D. Hutcheson, Craig J. Goergen, Frederick J. Schoen, Masanori Aikawa, Peter Zilla, Elena Aikawa, Glenn R. Gaudette

**Affiliations:** ^1^Department of Biomedical Engineering, Florida International University, Miami, FL, United States; ^2^Weldon School of Biomedical Engineering, Purdue University, West Lafayette, IN, United States; ^3^Brigham and Women's Hospital, Harvard Medical School, Boston, MA, United States; ^4^Chris Barnard Division of Cardiothoracic Surgery, University of Cape Town, Cape Town, South Africa; ^5^Worcester Polytechnic Institute, Worcester, MA, United States

**Keywords:** cardiovascular medicine, heart transplant, arterial disease, aortic valve, myocardial regeneration, tissue engineeering, interdisciplinary/multidisciplinary

## Abstract

The first successful heart transplant 50 years ago by Dr.Christiaan Barnard in Cape Town, South Africa revolutionized cardiovascular medicine and research. Following this procedure, numerous other advances have reduced many contributors to cardiovascular morbidity and mortality; yet, cardiovascular disease remains the leading cause of death globally. Various unmet needs in cardiovascular medicine affect developing and underserved communities, where access to state-of-the-art advances remain out of reach. Addressing the remaining challenges in cardiovascular medicine in both developed and developing nations will require collaborative efforts from basic science researchers, engineers, industry, and clinicians. In this perspective, we discuss the advancements made in cardiovascular medicine since Dr. Barnard's groundbreaking procedure and ongoing research efforts to address these medical issues. Particular focus is given to the mission of the International Society for Applied Cardiovascular Biology (ISACB), which was founded in Cape Town during the 20th celebration of the first heart transplant in order to promote collaborative and translational research in the field of cardiovascular medicine.

## Introduction

**Figure 1:**
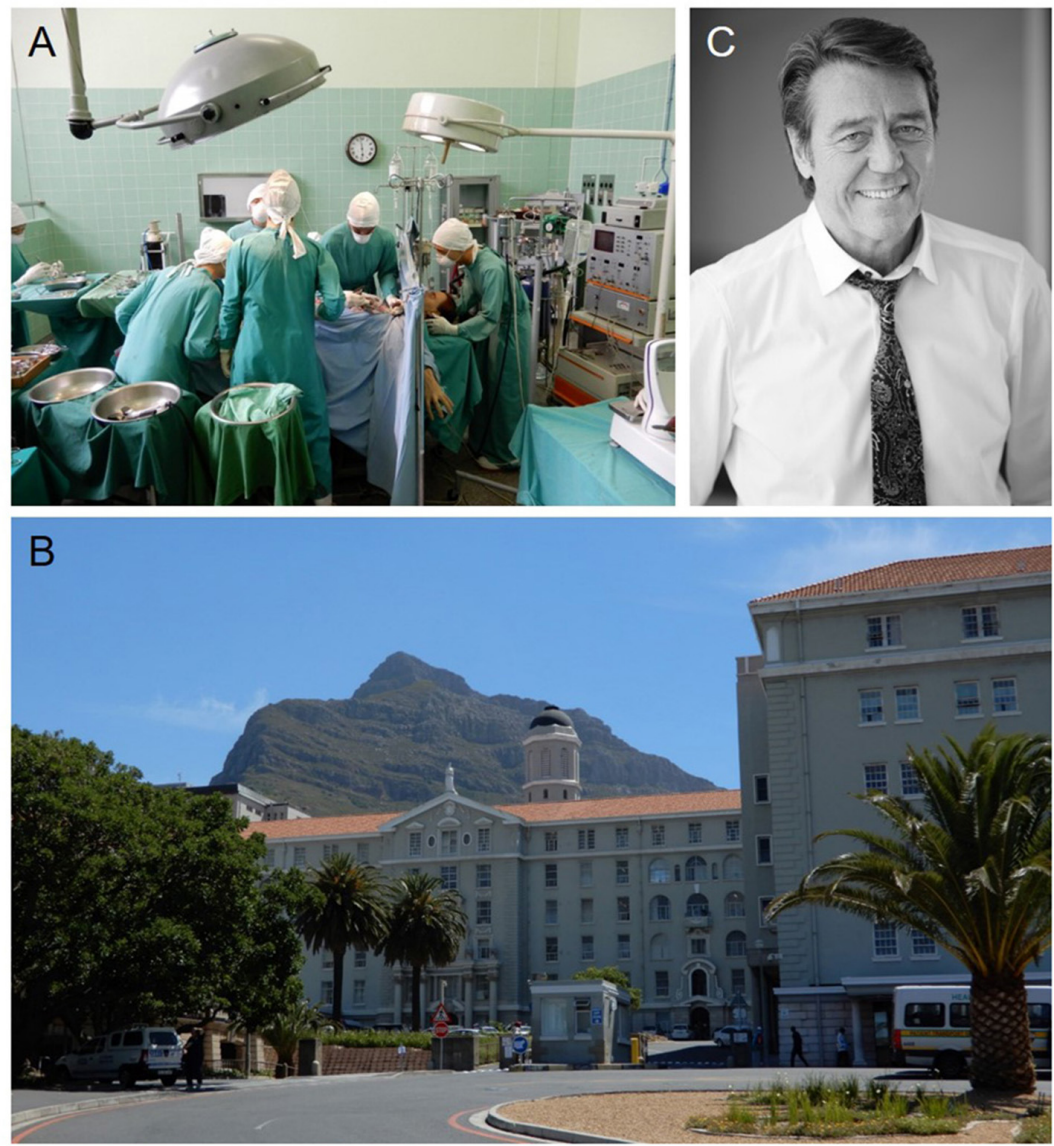
**(A)** Wax figures of the cardiac surgeon Christiaan Barnard, his team, and the patient Louis Washkansky during the first human heart transplantation at the Heart of Cape Town Museum in Groote Schuur Hospital that took place in 1967. **(B)** Groote Schuur Hospital where the first human heart transplantation was performed by Christiaan Barnard. This beautiful hospital is located on the slope of Devil's Peak shown in the background. **(C)** Peter Zella, MD, PD, Ph.D., FCs, Head of the Christiaan Barnard Department of Cardiothoracic Surgery at Groote Schuur Hospital of the University of Cape Town. He co-founded the ISACB and was a past president of the society. Dr. Zilla organized the 50th Anniversary Heart Transplant Celebration in Cape Town, “Courage and Innovation: 50 Years of transplantation” at Groote Schuur Hospital in December, 2017.

Christiaan Barnard, an innovative surgeon, transplanted the world's first human heart on December 3, 1967 in Cape Town, South Africa (Figures 1A,B). Soon after, surgeons across the world started transplanting hearts into patients with end-stage heart disease. The potential of rejection required immunosuppression, which left patients susceptible to infection. The approval of cyclosporine use for transplant recipients allowed for better post-transplant patient care and improved patient survival. In 2016, 3,209 hearts were transplanted in the U.S. alone and over 5,000 worldwide (1). However, the availability of transplantable hearts and their function once implanted is still far from optimal. To help overcome issues in the field of cardiac and vascular diseases more broadly, a collaborative group of cardiac surgeons, cardiologists, engineers and biologists founded the International Society for Applied Cardiovascular Biology (ISACB) in Cape Town during the 20th celebration of the first heart transplant in 1987. Now after more than 30 years, ISACB has nurtured an alliance among academic scientists and engineers, clinicians, and industry-based scientists to understand, prevent, and manage cardiovascular disease.

While scientists, engineers and clinicians have a long history of cooperation, with strong academic roots and participation in professional societies, the participation of industry in professional society meetings and has been dominated by marketing considerations. Proprietary concerns have further isolated many corporate scientists from open forums of communication. As stated by founding member and former ISACB President Peter Zilla, these “traditional roles and stereotypes must rapidly wane in light of the complexity that is required for any biologically “conscientious” product of today or tomorrow.…and…industry scientists must be better integrated within the academic and medical communities.” Dr. Zilla (Figure 1C), a surgeon scientist and Head of the Christiaan Barnard Department of Cardiothoracic Surgery of the Groote Schuur Hospital and the University of Cape Town, emphasizes the importance of understanding relevant science and corporate considerations while providing the surgeon with a usable solution as essential in developing better treatments for cardiovascular diseases. Collaborations between scientists, surgeons, engineers and investigators from other fields stimulates new opportunities to develop translatable solutions to significant cardiovascular issues. Thirty years later, and now 50 years after the world's first heart transplant, ISACB continues to foster a multidisciplinary convergence of professional expertise and experiences, through the *application* of biology to clinical medicine in order to prevent and overcome cardiovascular disease. Moreover, ISACB has also fostered collaboration among professional societies focused on cardiovascular biology, cardiology, surgery, pathology, and bioengineering, including joint meetings with the Society for Cardiovascular Pathology (SCVP), the North American Vascular Biology Organization (NAVBO), Heart Valve Tissue Engineering (HVTE), and others.

**Figure 2:**
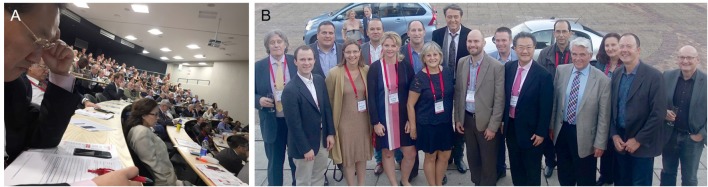
**(A)** ISACB Meeting in 2017 took place as part of the 50th Anniversary Heart Transplant Celebration in Cape Town, “Courage and Innovation: 50 Years of transplantation” at Groote Schuur Hospital. **(B)** ISACB members at the reception of the 50th Anniversary Heart Transplant Celebration in Cape Town, “Courage and Innovation: 50 Years of transplantation”.

In this perspective, we summarize developments in the restoration of cardiac function, including improved blood flow, valvular repair, replacement and tissue engineering, regeneration of myocardial tissue, mechanisms for vascular and valvular diseases, and other related areas. Particular attention will be paid to the practical application of potential therapies, as was discussed at the scientific sessions during the 30th anniversary of ISACB, which was held in Cape Town in December of 2017 to coincide with the celebration of the world's first heart transplant (Figure 2). More specifically, ISACB members have made important (and largely ongoing) advances and contributions to:

1. Unraveling *the mechanisms of atherosclerosis and its complications* (such as myocardial infarction), coupled with imaging technologies that reveal dynamic vascular and cardiac structures, atherosclerotic risk factors, and improved diagnostic strategies. This mechanistic understanding has immense clinical benefit. Recent major areas in atherosclerosis research that have made remarkable progress include the biology of vascular inflammation, assessment of vulnerable plaque, and advancements in lipid lowering statins.
2. Leading a virtual explosion in the number and scope of *cardiovascular surgical and interventional diagnostic and therapeutic procedures and devices used to manage heart disease. Four* developments are noteworthy in this regard: (1) the emergence of pediatric and adult cardiac surgery as routine therapies, including repairs for congenital cardiac abnormalities and acquired valvular heart disease, and valve replacement and coronary artery bypass surgery, (2) the growth of cardiac transplantation as a clinically-important therapeutic modality, beginning in 1967, and enabled by the development of endomyocardial biopsy as a primary and invaluable diagnostic tool, and the widespread use of this technology in patients with diverse pathologies of the myocardium; (3) the development and use of a broad array of prosthetic and adjunctive medical devices (including heart valves, vascular grafts and stents, and cardiac assist devices), demonstration of their complications, and improved generations of these devices, often through collaborations with industry; and (4) the recognition of the central importance of myocardial protection in cardiac surgery and intervention, which permitted the above to occur.
3. *Elucidating the impact of genetic abnormalities on many specific subsets of cardiovascular disease*, including the single-gene mutation etiologies of congenital abnormalities, (hypertrophic, dilated, and arrhythmogenic right ventricular) cardiomyopathies, channelopathies, and connective tissue disorders such as Marfan, Loeys–Dietz, and Williams syndromes, as well as complex multi-gene phenotypes and gene-environment interactions.

We describe below selected areas of current interest and active contribution of ISACB members that were discussed at the Cape Town 50th Anniversary meeting that are likely to yield considerable clinical benefit over the next several decades.

## Vascular Disease, Arterial Remodeling, and Vascular Replacement

Vascular disease encompasses a broad range of pathologies, extending from the cerebral vasculature to vessels in the lower limbs. While there is a broad range of arterial and venous diseases with varying risk factors, symptoms, and complications, this section focuses on two of major current clinical issues: atherosclerosis and aneurysms. Several recent studies and findings provide an overview of current efforts and areas for future work.

### Atherosclerosis and Tissue Engineered Vascular Grafts

Coronary artery atherosclerosis is associated with an inflammatory process (2) and contributes to significant morbidity and mortality (3). Surgeons often implant bypass grafts to deliver oxygenated blood around a stenosis to distal coronary beds. Current gold standard treatments use vessels harvested from other parts of the body since this autologous approach outperforms synthetic grafts. Unfortunately, these vessels require surgical harvesting and are prone to restenosis due to intimal hyperplasia. A current clinical focus is the development of a long-lasting tissue-engineered vascular graft (TEVG) (4). Despite significant efforts, ideal TEVGs have remained elusive. Many groups are working on developing TEVGs with appropriate mechanical properties, bioactivity, and biocompatibility (4, 5). Protein-coated polytetrafluoroethylene (ePTFE) grafts lined with autologous endothelial cells have shown long-term patency in almost 500 patients. The complexity of the cell sourcing and seeding procedures, however, does not make this technique amenable to routine use in vascular surgery. Yet, the clinical successes indicate the potential at this early stage of tissue engineering efforts. Ongoing efforts *in vitro* and *in vivo* seek to optimize long-term patency, mechanical properties, and reendothelialization (6). Recent advancements suggest continued improvements are possible and continued development could eliminate many of the issues associated with current synthetic grafts.

Atherosclerosis can also be present in peripheral arteries, and peripheral artery disease (PAD) can lead to intermittent claudication and critical limb ischemia in later stages of disease progression (7). The risk of developing lower-limb PAD increases with obesity, a history of atherosclerosis, high triglycerides, low high-density lipoprotein, and aging (8). Ongoing efforts seek to develop non-invasive interventions to treat atherosclerosis and prevent deleterious remodeling of the vascular wall. A recent study showed an association between serum levels of sortilin, a glycoprotein involved in glucose and lipid metabolism, with aortic calcification and general cardiovascular disease risk (9). Carotid artery atherosclerosis revealed PCSK6 as a novel protease, possibly making these lesions prone to rupture (10). The development of a novel platelet lysate hydrogel has shown promise to promote angiogenic activity of mesenchymal stem cells (MSC) that can also be delivered concomitantly (11). While each of these individual findings may lead to a therapeutic breakthrough, the combination of multiple studies over the next 50 years has the potential to improve our mechanistic insight into atherosclerosis and PAD, providing unique treatment solutions.

### Emerging Evidence for Monocyte/Macrophage Heterogeneity

Accumulating evidence from basic science and clinical medicine suggests that inflammation plays critical roles in the pathogenesis of atherosclerotic vascular diseases and their clinical complications (12). Emerging evidence indicates that macrophages are a heterogeneous population (13). Similarly, we know that monocytes, generally considered as macrophage precursors, are also heterogeneous (14). Changes in macrophage behavior and attributes in response to systemic or local environmental cues may help execute specific functions during the disease process. Traditionally macrophages were thought to adopt a pro-inflammatory or anti/non-inflammatory phenotype in response to stimuli (M1 vs. M2 polarization), but new evidence suggest that macrophage heterogeneity is more multi-dimentional (15–17). Studies using single cell analyses have demonstrated the dynamic and complex nature of human primary monocytes and macrophages heterogeneity (18–20). Understanding the underlying mechanisms of monocyte/macrophage heterogeneity and related therapeutic implications may require innovative approaches such as machine learning from large clinical studies.

### Aneurysms: Imaging, Biomechanics, and Novel Therapies

Abdominal aortic aneurysm (AAA) is an inflammatory disease of the aorta resulting in pathologic dilation of the vessel wall. Clinically, an aortic diameter 50% larger than normal is considered aneurysmal, and only surgical treatment options currently exist (21). Between 5 and 10% of people in the industrialized world over the age of 65 suffer from AAAs (22, 23), accounting for roughly 16,000 deaths and 150,000 inpatient hospitalizations per year in the U.S (24, 25). Although recent studies have provided insight into the pathogenesis of AAA, a detailed understanding of the underlying mechanisms that lead to AAA expansion remains incomplete.

Development of novel therapies that will interrupt development of an AAA or halt aneurysm progression remains a challenge (26). Efforts are focusing on investigating the association between genetic variants and aneurysm formation (27) and the role of enzyme activity in extracellular matrix (ECM) changes within the aortic wall (28). Further work has focused on the role of the inflammasome, including both innate immunity and inflammation, in aneurysm formation and progression (29). Recent studies have shown that serum amyloid A, a protein that associates with high-density lipoprotein when in circulation, exacerbates acute vascular events by activating the inflammasome (30, 31). Others have investigated the correlation between circulating biomarkers and aortic disease, showing that elevated circulating levels of the soluble receptor for advanced glycation end products is associated with a variety of aortopathies, independent of aortic diameter (32). Identifying patients at increased risk for aneurysm development and then increasing aortic wall strength through pharmacologic means could slow growth of AAAs to large diameters where rupture is more likely.

Beyond aortic wall research, blood flow hemodynamics have been shown to be critical to the formation and growth of aneurysms, dissections, and thrombus (33). This provides strong motivation to develop sophisticated data-driven models of blood flow, pressure, and wall elasticity associated with AAAs. Recent work focused on implementing a multi-modality imaging approach that combined high frequency ultrasound (US) and optical coherence tomography (OCT) as inputs for a murine computational modeling study (34). The results showed that differences in final lesion size and compositions correlated with vortical structures obtained through mouse-specific fluid dynamic simulations, suggesting that differences in morphology and hemodynamics play crucial roles in AAA formation. These data agree with a large amount of previous work where imaging-based computational findings have suggested a link between hemodynamic perturbations and aneurysmal disease heterogeneity (35). The combination of imaging, hemodynamic simulations, and biomechanical analysis is proving to be useful for exploring potential translational strategies that could soon be useful to predict possible aneurysm expansion and rupture (36, 37). Taken together, these recent advancements suggest a bright future for multi-disciplinary cardiovascular research in clinical medicine, genetics, biology, and engineering to address unmet clinical need associated with AAA.

## Valve Disease and Valve Repair and Replacement Technologies

Valve diseases constitute a global health burden. In developed countries, age-related calcific aortic valve disease (CAVD) eventuates in aortic stenosis, whereas in developing countries, rheumatic heart disease remains the leading cause of valvular structural abnormalities (38). Other key causes of valvular dysfunction include mitral valve prolapse (myxomatous valve disease) and functional mitral regurgitation owing to ischemic heart disease. High rates of congenital valve abnormalities present complications in pediatric patients without regard to environmental conditions. Each of these causes of valve dysfunction represent unique challenges in the management of valve disease, but appropriate solutions hinge on understanding the factors that govern valve homeostasis and function.

Although decades of basic and clinical research and the advent of lipid lowering therapies (especially statins) have markedly reduced morbidity and mortality associated with atherosclerotic cardiovascular diseases, clinical trials have shown that statins have no effect on progression of existing CAVD, and thus no effective therapy is available. As a result, clinical options for patients with CAVD are limited to invasive open heart surgery or transcatheter valve implantation (39).

Pathological remodeling most commonly affects the aortic and mitral valves, likely a consequence of higher systemic pressures, underscoring the importance of biomechanical function and sensitivity. Given the relatively high incidence and severity, we focus our discussion here on aortic valve disease and replacement, an area of tremendous clinical need.

### Aortic Valve, Function, Structure, Biology, and Target Discovery

Unidirectional blood flow from the left ventricle to the aorta for systemic distribution normally occurs through coordinated action of three leaflets. Leaflet action is controlled by a layered and highly organized ECM microarchitecture (40–43). The ECM structure is maintained by two cell populations: valvular endothelial cells (VECs) and valvular interstitial cells (VICs). VECs appear phenotypically distinct from other endothelial cell populations in vascular tissues and exhibit regional heterogeneity with side-specific differences in gene expression (44–46). VICs are a poorly defined population of cells with subpopulations of fibroblasts, myofibroblasts, smooth muscle cells, and neuron-like cells previously identified within the leaflets (47, 48). Phenotypic changes in VECs and VICs have been associated with aortic valve remodeling (49), but the relative contributions of these cells and associated subpopulations remain unknown. The role of inflammation in valve remodeling (50, 51) is especially relevant when considering approaches to valve disease in developing countries, where rheumatic heart disease is a major contributor. VECs and VICs also display mechanosensitivity and readily respond to changes in the mechanical environment (52–55). The complex cellular and biomechanical environment is difficult to recapitulate *in vitro* and animal models of aortic valve disease are lacking (56), making mechanistic studies on the biomechanical and biochemical initiators of disease difficult to perform.

Recent studies have sought to overcome this limitation by using large, unbiased proteomic and transcriptomic approaches to characterize molecular changes in aortic valve leaflets obtained from patients undergoing replacement surgeries (57). Combining pathological characterization of the leaflets following resection with network-based analysis of the proteomic and transcriptomic data has yielded new insight into the potential molecular drivers of aortic valve disease. Coupled with new genome wide association studies that have revealed new lipid associations with aortic valve disease, these big data approaches may provide new clues about points of non-invasive therapeutic intervention and the development of drug-based therapies (58, 59). However, challenges remain in identification of patients during the early stages of disease before gross remodeling of the aortic valve leaflets necessitate replacement.

### Synthetic and Bioprosthetic Approaches to Aortic Valve Replacement

Given the lack of non-invasive treatment or suitable options for CAVD, the traditional clinical approach has been surgical valve replacement. First introduced in 1960, early iterations of devices for aortic valve replacement utilized mechanical valves consisting of caged-ball or tilting disk designs surgically implanted into the aortic orifice following removal of the diseased valve (60). These devices provided the first viable clinical solution for patients with aortic valve abnormalities and offered extraordinary reduction in mortality associated with CAVD. Of note, inoperable patients with CAVD have a 2–3 year mortality of < 50% (61, 62). Though these devices helped correct valve dysfunction, nearly all patients who received mechanical valves suffered valve-related complications within 10 years, and many died of these complications (63).

To enhance biocompatibility and create a more normal geometry, bioprosthetic valves were introduced in the clinic in the late 1960s as an alternative to mechanical valves (64). Bioprosthetic valves are fabricated from glutaraldehyde treated (and hence non-viable) porcine aortic valve or bovine pericardial tissue formed into a tri-leaflet structure. Bovine pericardium is used most frequently today. These valves do not require lifelong anticoagulation therapy, and bioprosthetic valves more adequately recapitulate the biomechanics and hemodynamics of native aortic valves. Nevertheless, bioprosthetic valves frequently undergo calcification, leading to stenosis or tearing with regurgitation. After ~15–20 years, bioprosthetic valves often must be replaced, requiring the patient to undergo an additional invasive surgical procedure. The mineral forms due to phosphorus in devitalized cell remnants and possibly residual aldehyde affinity for mineral. Newer versions of bioprosthetic valves overcome this limitation through detergent-based treatments that reduce cell-based material and inhibit mineral deposition (65). To avoid multiple surgeries, modern mechanical valves have been deemed more suitable for younger patients who need aortic valve replacement. Clinicians must weigh the relative risks of reoperation to replace bioprosthetic valves vs. the risks associated with anticoagulant therapy in patients with mechanical valves (66).

The advent of transcatheter aortic valve implantation (TAVI) has begun to revolutionize aortic valve replacement. Synthetic or bovine pericardial-based aortic valves are placed into the aortic annulus using an endovascular catheter. The catheter is most often introduced through the femoral artery and guided to the annulus whereupon the replacement aortic valve is deployed, displacing the diseased aortic valve (67). First introduced for elderly patients and those deemed unfit for surgical-based replacements, TAVI is becoming standard care for many patients with CAVD (68). Patients undergoing TAVI procedures have similar outcomes as those who receive surgical aortic valves (69). Early analyses indicated that TAVI may induce stroke, paravalvular leak, and vascular wall damage during catheterization; however, subsequent studies have shown that other complications may be less of a concern than those arising from surgery (70, 71). TAVI “valve-in-valve” approaches also obviate the need for open surgical procedures for patients with degeneration of a previously implanted bioprosthetic valve. After the initial bioprosthetic valve deteriorates, a TAVI procedure can introduce a new valve that is likely to exceed the expected lifespan of the patient.

The leading cause of aortic valve disease in developing countries is rheumatic heart disease, but the local infrastructure is not generally well-suited for open heart procedures. TAVI may provide a more appropriate option for patients in these regions (72); however, two specific limitations must be overcome. Typically, aortic valve disease and bioprosthetic degeneration are associated with the deposition of calcific mineral on the leaflets. This mineral provides a structure to anchor TAVI valves, but rheumatic-induced aortic valve remodeling does not usually involve heavy calcification. Positioning the catheter during TAVI also requires imaging modalities not commonly available in developing countries. Recently developed TAVI strategies designed specifically for low resource settings may help overcome these limitations (73). The new design employs a supra-annular anchoring technique that latches to the non-calcified valve structure and provides tactile feedback that allows the clinician to locate the correct annular position without the need for fluoroscopic imaging. This technique could address a major unmet clinical need in developing countries.

### Engineering Living Aortic Valve Tissue

Since the first replacement aortic valves were introduced, advancements in both valve design and replacement techniques have provided lifesaving options for many patients. However, issues remain, particularly for pediatric patients who require aortic valve replacement due to congenital valvular abnormalities. These children often require multiple procedures to replace valves that do not adapt to somatic growth, and calcification of valves and conduits is accelerated in young recipients. These patients would benefit from engineered aortic valve constructs that fully integrate with native host tissues, do not degenerate, and adapt to size and pressure changes in the cardiovascular system. Efforts to develop tissue engineered aortic valves should integrate knowledge of the complex biological environment, dynamic biomechanics, material durability, and delivery/implantation methods discussed in the previous sections. Early attempts to engineer living aortic valve tissues employed biodegradable scaffolds seeded with mixed populations of arterial-derived endothelial cells and fibroblasts (74). These constructs yielded ECM deposition consistent with native valve structure after 2 months in an ovine model, demonstrating the potential utility of a living engineered tissue that can actively remodel appropriately after implantation (75).

Translation of these proof-of-concept techniques to clinical practice for human patients remains elusive, however. Questions persist on the appropriate cell source, the most appropriate material for the scaffolds, the minimum biomechanical functionality required for implantation, and the methods to assess remodeling *in situ* following implantation (76). In the early 2000s, the first tissue engineered aortic valve replacement surgeries were performed in neonates with severe congenital malformations (77). These valves exhibited beneficial early remodeling in an ovine model, and gross long-term ECM remodeling was attributed to a problem with the animal model. The outcomes from initial clinical trials, however, were largely poor. Many of the valves exhibited remodeling concomitant with inflammation, including fibrosis and deterioration, comparable to the observations made in the ovine endpoints (77).

These early outcomes reduced enthusiasm for aortic valve tissue engineering, but the clinical need for pediatric patients with aortic valve dysfunction remains. Early clinical successes have been noted in pediatric mitral valve repair using constructs of porcine small intestinal submucosa handmade in the clinic to resemble valve leaflets (78). The *ad hoc* use of this material in patients with few other clinical options has yielded promising results in short-term clinical follow-ups and work by recruiting endogenous cells that stimulate leaflet remodeling and growth (79, 80). Similar strategies are being developed for aortic valve replacement. Many approaches currently in pre-clinical development seek to recruit host cells after implantation of a polymer matrix without cells or other biological adjuncts. In such an approach, proper ECM development and leaflet maturation takes cues from and depends on processes that occur in native valve development (so-called “*in situ* tissue engineering”) (81). This strategy enables off-the-shelf availability of constructs without the need for maintenance of cellular viability, and could provide a clinically feasible solution that avoids cell sourcing complications (82).

Whether these tissue engineered constructs can adequately recapitulate the function of native aortic valves remains to be seen. Perhaps the complete recapitulation of the complex biological structure and biomechanical properties of native valve are not required to produce adequate and lasting function. Imperfect strategies that offer new life to a patient with no other options provide clinical value, and knowledge gained through iterations of incremental improvement will help fill gaps in our current understanding of aortic valve biology and function. Ultimately, non-invasive therapeutics may prevent or reverse adult-onset aortic valve remodeling, and minimally invasive implantation of tissue engineered valves may fix congenital abnormalities in pediatric patients. Achieving these goals will require concerted interdisciplinary efforts of basic scientists, engineers, and clinicians. All are well-represented within ISACB.

## Cardiac Regeneration

Unlike other tissues in the body, the heart does not possess significant regenerative capacity. The adult heart responds to infarction by creating a collagen dense scar. While this may strengthen the mechanical properties of the wall to help eliminate ventricular rupture, it decreases the overall pump capacity of the heart. In many cases, this decreased function leads to congestive heart failure. While a heart transplant is currently the only “tried and true” means to restore mechanical pump function in these patients, the lack of organ donors calls for additional solutions.

Engineered cardiac tissues offer a potential solution. Rapid advances in cell therapy, including induced pluripotent stem cells, have demonstrated that cells can be grown in the laboratory and differentiated into cardiac muscle cells. In order to restore contractile function in the heart, these cells need to form an aligned and synchronized dense network, and a vascular supply will be needed to maintain viability. Thus, tissue engineered cardiac scaffolds should provide for cell attachment and survival while allowing the scaffold to contract in sync with the rest of the heart.

### Engineering Cardiac Scaffolds

When considering scaffolds for cardiac tissue engineering, many factors must be considered for the vast applicability of these materials (83). Cost, sustainability, and labor skill requirements must all be considered for widespread use. A potential starting point for engineered cardiac tissue is an acellular scaffold (84). Investigators have used a master bank of human cells to produce the ECM for these scaffolds. This allows for a controlled initial material source, which helps bring costs down and maintain quality control over the product. Decellularization leaves an ECM that is attractive for native cells to adhere and proliferate (85–87). Lyophilization and sterilization yields an “off the shelf” product, which also helps bring down costs. These scaffolds can be produced in a Good Manufacturing Practices (GMP) facility with appropriate quality controls, allowing for consistent production of scaffolds with the same properties. Most pre-clinical work to date with this scaffold has been in a congenital model, showing that the scaffold grows with the animal. The Emmert/Hoerstrup group is currently working to develop the scaffold as a cardiac patch.

Difficulties remain in vascularizing scaffolds to maintain cell viability. Instead of using mammalian cells to produce a scaffold, investigators are looking toward the plant kingdom, specifically spinach leaves (88). After the decellularizing process, the vascular network inherent to the plant remains and can be used to perfuse fluid. Microspheres, of similar size to red blood cells, were also able to pass through the plant vasculature, and the scaffold is able to serve as a basement membrane for contracting cardiac myocytes. Further work, however, is required for clinical realization of this technique.

### Cells for Cardiac Regeneration

Clinical trials on cell therapy for heart disease have demonstrated only limited success (89). This may be due in part to the variability in the cells used in cardiac cell therapy. Most cardiac clinical trials have utilized MSC. While this cell type has not demonstrated deleterious effects, improvement in cardiac function appears to be limited.

Embryonic stem cells (ESCs) and induced pluripotent stem cells (iPS cells) have demonstrated the potential to form contractile myocytes. ESCs can proliferate to provide a plentiful source of cells. They are also able to differentiate into contracting cells with many properties similar to adult cardiac myocytes. ESCs, however, remain a topic of controversy. An exciting discovery in 2006 introduced a new cell type—iPS cells that can be produced from adult differentiated cells (e.g., fibroblast) through genetic engineering (90). By inserting specific genes regulating transcription factors, the adult cells can be induced to becoming an embryonic-like stem cell. These cells can then be differentiated into contractile cells with properties similar to cardiac myocytes. Thus, a patient's own cells can potentially yield cardiac myocytes that restore cardiac function, eliminating any immune rejection response from the recipient. However, significant concerns still remain for both ESCs and iPS cells prior to their use in the clinic. Cell sorting and validation is essential to moving the field forward. Incorporation of the wrong cell type in the heart can lead to fatal arrhythmias or worse, and proliferation of these cells must be regulated.

## The Next 50 Years

Despite decades of active research efforts in cardiovascular biology, few basic science discoveries have arrived in the clinic as efficient drugs or devices. Indeed, many preclinical breakthroughs have failed to survive clinical translation. Because of insufficient expertise and tight funding, academic investigators often struggle to translate findings into clinical development (91, 92). This gap also results from strategies in industry to avoid investing in early, high-risk targets (93, 94). Clearing such roadblocks requires new paradigms for translational research. As ISACB has consistently promoted since it was founded, close collaboration between academic investigators and industry scientists, who can share clear goals and understand potential mutual benefits, will facilitate exchange of ideas, resources, and expertise and lead to innovative therapies for cardiovascular diseases (15, 95).

Looking back on the improvements made in treating cardiovascular diseases over the past 50 years, one cannot help but wonder: what key advances will occur in the next 50 years? In addition to the areas discussed above, endovascular therapies, valve repair and replacement technologies, arrhythmia ablation, xenotransplantation, and long-term cardiac support (both mechanical and biological) will almost certainly continue to improve. Additionally, it is probable that significant strides will be made toward directed prevention of a broad range of cardiovascular conditions. New discoveries require innovative technologies. Considering the accelerated speed of technological development, courage and innovation are important values, as suggested during the 50th Anniversary Heart Transplant Celebration. With effective collaboration fostered by the ISACB and similar cross-disciplinary societies, the next 50 years will likely lead to many more life-saving treatments that will hopefully be extended to ALL patients around the world.

## Author Contributions

JH, CG, FS, MA, PZ, EA, and GG all contributed to the text and editing of the manuscript.

### Conflict of Interest Statement

The authors declare that the research was conducted in the absence of any commercial or financial relationships that could be construed as a potential conflict of interest.
